# Supplementary material for: EFFICHRONIC study protocol: a non-controlled, multicentre European prospective study to measure the efficiency of a chronic disease self-management programme in socioeconomically vulnerable populations
Source: BMJ Open. 2019 Dec 18;9(12):e032073. doi: 10.1136/bmjopen-2019-032073 (PMC6937023; doi:10.1136/bmjopen-2019-032073)
Supplement: Author's manuscript [file bmjopen-2019-032073.draft_revisions.pdf]

BMJ Open

BMJ Open is committed to open peer review. As part of this commitment we make the peer review history of every article we publish publicly available.

When an article is published we post the peer reviewers' comments and the authors' responses online. We also post the versions of the paper that were used during peer review. These are the versions that the peer review comments apply to.

The versions of the paper that follow are the versions that were submitted during the peer review process. They are not the versions of record or the final published versions. They should not be cited or distributed as the published version of this manuscript.

BMJ Open is an open access journal and the full, final, typeset and author-corrected version of record of the manuscript is available on our site with no access controls, subscription charges or pay-per-view fees (http://bmjopen.bmj.com).

If you have any questions on BMJ Open's open peer review process please email info.bmjopen@bmj.com

BMJ Open

EFFICHRONIC Study protocol: a multicenter European Chronic Disease Self-Management Programme in socio-economic vulnerable population

Journal:	*BMJ Open*
Manuscript ID	bmjopen-2019-032073
Article Type:	Protocol
Date Submitted by the Author:	31-May-2019
Complete List of Authors:	Boone, An LD; Gobierno del Principado de Asturias Consejeria de Sanidad<br>Pisano-Gonzalez, Marta ; Health Service of the Principality of Asturias<br>Valsecchi, Verushka; Centre Hospitalier Universitaire de Montpellier<br>Tan, Siok; Erasmus Medical Center<br>Pers, Yves-Marie; Centre Hospitalier Universitaire de Montpellier<br>Vazquez-Alvarez, Raquel; Health Service of the Principality of Asturias<br>Peñacoba-Maestre, Delia; Health Service of the Principality of Asturias<br>Baker, Graham; QISMET<br>Pilotto, Alberto; Ente Ospedaliero Ospedali Galliera<br>Zora, Sabrina; Ente Ospedaliero Ospedali Galliera<br>Raat, Hein; Erasmus Medical Center<br>Hevia-Fernandez, Jose Ramón; Gobierno del Principado de Asturias Consejeria de Sanidad
Keywords:	Chronic Disease, Social Determinants of Health, Caregivers, Patient participation, Self-Management, Cost-efficiency

SCHOLARONE™
Manuscripts

TITLE PAGE

Title

EFFICHRONIC Study protocol: a multicenter European Chronic Disease Self-Management Programme in socio-economic vulnerable population.

Authors

An LD Boone (corresponding author), Government of the Principality of Asturias, Ministry of Health, C/ Ciriaco Miguel Vigil, 9, 33006 Oviedo – Asturias, Spain, An.Boone@asturias.org, phone: +34 985 10 68 73, fax: +34 985 10 65 20.

Marta M Pisano-González, SESPA, Health Service of the Principality of Asturias; Oviedo, Spain.

Verushka Valsecchi, Unité d'Immunologie clinique et Thérapeutique ostéo-articulaire, Centre Hospitalier Universitaire (CHU) de Montpellier; Montpellier, France.

Siok-Swan Tan, department of Public Health, Erasmus MC University Medical Center; Rotterdam, the Netherlands.

Yves-Marie Pers, Unité d'Immunologie clinique et Thérapeutique ostéo-articulaire, Centre Hospitalier Universitaire (CHU) de Montpellier; Montpellier, France.

Raquel Vazquez-Alvarez, SESPA, Health Service of the Principality of Asturias; Oviedo, Spain.

Delia Peñacoba-Maestre, SESPA, Health Service of the Principality of Asturias; Oviedo, Spain.

Graham Baker, Quality Institute for Self Management Education & Training (QISMET); Portsmouth, United Kingdom.

Alberto Pilotto, department Geriatric Care, E.O. Galliera Hospital; Genova, Italy.

Sabrina Zora, department Geriatric Care, E.O. Galliera Hospital; Genova, Italy.

Hein Raat, department of Public Health, Erasmus MC University Medical Center; Rotterdam, the Netherlands.

Jose Ramón Hevia-Fernandez, Government of the Principality of Asturias, Ministry of Health; Oviedo, Spain.

On behalf of the EFFICHRONIC consortium

Word count: 3861

Keywords: Chronic Disease, Social determinants of Health, Caregivers, Patient participation, Self-Management

For peer review only

Abstract

INTRODUCTION

More than 70% of mortality worldwide is due to chronic conditions. In addition, it has been proven that social determinants have an enormous impact on health-related behaviour and on the received attention from healthcare services, producing health inequalities. The objective of this study is to reduce the burden of chronic diseases in five European regions, focusing on vulnerable populations, and to increase the sustainability of health systems by implementing a chronic disease self-management programme (CDSMP).

METHODS AND ANALYSIS

2,500 people with a chronic condition or informal caregivers belonging to vulnerable populations will be enrolled in the CDSMP in five European regions in Spain, Italy, United Kingdom, France and the Netherlands. Inclusion of patients will be based on a geographical, socio-economic and clinical stratification process. The programme will be evaluated on self-efficacy, quality of life and cost-effectiveness by means of validated questionnaires at baseline and at 6 months from baseline.

ETHICS AND DISSEMINATION

This study will follow the directives of the Helsinki Declaration and will adhere to the EU General Data Protection Regulation (GDPR). The project's activities, progress and outcomes will be disseminated via promotional materials, use of mass media, online activities, presentations at events and scientific publications.

REGISTRATION DETAILS

The trial was registered at ISRCTN on 20/06/2018 with registry number 70517103.

For peer review only

Article Summary

Strengths and limitations of this study

- This study will gather valuable data on the CDSMP implementation in 5 different European countries with diverse cultural and socio-economic realities and distinctly organized health systems and regulations.
- Particularly, the recruitment strategies for the target hard-to-reach populations will provide important information on how to identify and select people with the greatest potential for reducing the burden of chronic diseases and to intervene effectively on them.
- Furthermore, the study will provide scientific data on the reliability of investment in chronic disease prevention.
- This study is not randomized controlled to not deprive any possible receivers of the benefits of the programme.
- It has been demonstrated that the positive results of these kinds of interventions reach their highest impact at one year post-intervention so ideally, a longer-term trial should be conducted.

For peer review only

Introduction

In the 21st century, chronic conditions are among the most important public health challenges mainly due to their double impact: the human suffering they cause and the damage they produce on the socioeconomic structure of countries. Although healthcare systems are still focusing on acute diseases[1], they are in full transformation because of demographic changes, the increasing prevalence of chronic diseases, the importance of lifestyle conditions, and the budgetary crisis of recent years. This shift requires new healthcare delivery models to ensure sustainability.

Non-communicable diseases (NCD) and fragile and vulnerable settings[2 3] are part of the ten threats to global health the World Health Organization (WHO) has warned of recently. More than 70% of mortality worldwide (41 million people each year), is due to chronic conditions, such as cancer, diabetes, respiratory and heart diseases. All of them are related to five main risk factors: tobacco use, physical inactivity, harmful consumption of alcohol, unhealthy diet and air pollution. Furthermore, 22% of the global population lives in vulnerable settings which exist in almost all regions of the world. As a result, the 13th WHO General Program of Work (2019 – 2023), expresses this particular concern with the slogan: "Promote health, keep the world safe, serve the vulnerable", urging precisely to work on health promotion in populations suffering particular vulnerability.[3]

The large impact of non-communicable diseases on national income is primarily due to loss of productivity by cause of absenteeism and inability to work, threatening the sustainability of recent macroeconomic achievements. Furthermore, there has been a steady global increase in healthcare expenditure on NCDs over the years. According to a systematic review on healthcare spending and national income, cardiovascular diseases account for 12% of healthcare expenditure in the European Union and diabetes related costs were estimated at 7%.[4] Besides, other conditions and risks factors such as depression or obesity are becoming more prevalent in higher-income EU countries and are greatly determined by poverty and low-income. [5 6] In addition, scientific evidence of recent decades has shown the enormous impact of social determinants, such as income, unemployment, housing quality, educational level or gender on health, manifested in very diverse health inequalities.[7] Socio-economic and cultural differences can produce short- and long-term inequalities concerning health-related and self-care behaviour[1], as well as the received attention from healthcare services. These observations confirm the 'inverse care law' theory described in 1971[8] by JT Hart, referring to the principle that the availability of good medical or social care tends to vary inversely with the need of the population served.

Fortunately, as evidence shows, chronic conditions can be prevented and controlled by reducing their risk factors.[9] These factors are mostly linked to unhealthy sedentary lifestyles and can be successfully modified by health literacy and thus, programmes that focus on self-efficacy and empowerment. [10 11]

The basis of the EFFICHRONIC project is the internationally acknowledged Chronic Care Model (CCM)[12] aimed to promote patients' empowerment and autonomy. The specific programme used in the project is the Chronic Disease Self-Management Programme (CDSMP).[13] It concerns a process-centered intervention constructed on the Social Learning Theory[14] and it is appropriate for any adult living with one or more chronic conditions. Since its beginning in 1990[15], the effectiveness of the CDSMP has been demonstrated by means of positive health outcomes and more efficient use of health resources.[16] The programme has a clear-cut community focus and is disseminated through an international infrastructure of training, certification and licensing.

Study Rationale

Research on prevention programmes has mainly been focusing on interventions targeted at a specific chronic condition. Furthermore, research on individuals with specific vulnerability profiles and data on the efficiency and cost-benefit of these programmes is scarce. This lack of data reduces, in some cases, the policy engagement and support; but most importantly, it prevents health systems from being more sustainable and efficient.[17 18]

EFFICHRONIC aims to empower individuals from deprived or socially excluded communities to manage their chronic condition, with an integrated approach and without being disease specific. The overall objective of the project is to reduce the burden of chronic conditions and increase the sustainability of health systems by implementing a chronic disease self-management programme in socio-economic vulnerable populations in five European regions.

Five specific objectives have been defined: 1) to identify vulnerable groups and individuals by means of a multidimensional analysis in the involved regions; 2) to design specific recruitment strategies to reach the targeted individuals and groups; 3) to implement the programme in the 5 regions with at least 500 individuals per region; 4) to generate a comprehensive framework for the impact assessment, including cost-efficiency and health-economic assessment; and 5) to define guidelines and policy recommendations to allow to the scaling up of the EFFICHRONIC methodology and intervention strategy to other regions in Europe.

For peer review only

Methods and analysis

Methodology

The methodology used for the community intervention of EFFICHRONIC is the “Chronic Disease Self-Management Programme" developed at Stanford University. The CDSMP is a public health intervention in the community provided by trained monitors. The programme is strongly evidence-based and consists of the following conceptual elements: self-efficacy, empowerment, salutogenesis, peer-to-peer education and vicarious training. It includes action planning and feedback about problem-solving skills and other competences such as the reinterpretation of symptoms and training in disease management.

A cascade training model is used in the CDSMP method. On top are Self-Management Resource Centre (SMRC) licensed T-trainers who possess the maximum teaching expertise, in charge of training Master Trainers, who in turn train monitors (or leaders) who will conduct the chronic disease self-management workshops. The monitors receive practical training on the philosophical and methodological basis of the CDSMP: empowerment, self-efficacy, group management, management of painful emotions, positive health, and efficient communication among other skills. Once trained, the monitors facilitate the workshops in pairs. In each country minimum 32 monitors will be trained to instruct 500 end-users: people with chronic conditions or caregivers. The CDSMP workshops have a duration of 6 weeks with sessions of 2,5 hours once a week in groups of 12 to 20 participants. The programme fidelity and quality plan include the supervision of workshops in the community by the master trainers by means of periodic protocolled monitor audits. Workshop assistants receive a handout with the workshop topics and homework assignments, a consultation book, and a certificate of attendance if they were present at minimum 4 of the 6 sessions.

Study design

EFFICHRONIC is a multicenter non-controlled prospective longitudinal study with a duration of 3 years in five European settings: the Principality of Asturias in Spain, the area of Genoa in Italy, several regions in the United Kingdom, the Occitanie region in France and the area of Rotterdam in the Netherlands. The participating countries represent a wide range of social, economic and health-related realities. The EFFICHRONIC intervention consists of the following phases: identification, recruitment and stratification of participants from vulnerable population groups, implementation of the CDSMP in the target population and an economic and efficiency evaluation with a pre-post design. Additionally, guidelines and policy recommendations will be developed based on the project conclusions to allow the scaling-up of the intervention to other contexts.

Study population

The study population will consist of 2,500 participants from the five previously described geographical areas. The target population involves two main groups: vulnerable people with a

For peer review only

chronic condition and informal caregivers who live socially isolated. The concept of vulnerability in EFFICHRONIC is adapted from a definition from the Spanish Red Cross which describes it as a situation between complete social inclusion and total exclusion.[19] Based on this definition, the inclusion criteria in EFFICHRONIC are characterized as follows: 1) having more than 65 years and living alone or in a nursing home; 2) being imprisoned (where possible); or 3) having difficulties to make ends meet, especially if belonging to an ethnic minority or being a legal immigrant. A chronic disease, self-reported or clinically evaluated, is outlined as a pathology listed in the International Classification of Primary Care (ICPC-2) with a code between 70 and 99, and should have more than six months of evolution. Informal caregivers are defined as persons taking care of someone with an illness, usually a family member, without receiving payment. They don´t have to suffer from a chronic condition themselves, but must comply with the vulnerability condition of isolation, such as (but not only) not having means of transportation, internet access or social support.

Besides the before-mentioned inclusion criteria, participants must be minimum 18 years old, reside in the selected geographical areas, have their basic housing needs met and possess an adequate knowledge of the local language. They should not be going through a period of crisis (e g being evicted), be diagnosed with severe mental health problems that cause distorted perception of reality and/or inability to function in group dynamics, have cognitive decline (e.g. Alzheimer´s), nor have active addictive disorders (Figure 1).

Sample selection and recruitment

The potential participants are being identified by means of a geographical, socio-economic and clinical stratification process. A distinction is made between the individual and social dimension of vulnerability, leading to different recruitment strategies that will be combined to be more efficient. First, geographical mapping of areas with a higher percentage of vulnerable population was done based on existing national and local indexes in all participating countries. Consecutively, individualized recruitment will take place in these areas by means of specifically designed actions. Multiple recruitment channels are used in coordination with local agents from different sectors, such as the health system, social services or associative and community-based structures. More specifically, the following profiles and organizations were involved in the recruitment process: health professionals, pharmacists, social workers, NGOs such as Caritas or the Red Cross, city councils and local associations. Due to the differences in organizational, social and health system structures of the involved study sites, recruitment strategies were adapted to the local circumstances of each of them.

Stratification

To identify people who will benefit most from the programme, individual socioeconomic and clinical parameters will be determined by a multidimensional stratification analysis. The tool used for this purpose is called the SELFY-MPI and is based on the previously validated Multidimensional Prognostic Index (MPI).[20] The self-administrable instrument was specifically designed for EFFICHRONIC and incorporates socio-economic and educational parameters.

For peer review only - http://bmjopen.bmj.com/site/about/guidelines.xhtml

Several vulnerability parameters will be extracted from the multidimensional analysis based on data on education, income, co-habitation, age, and social support.[21]

Implementation

Once the target population is identified and recruitment is ongoing, the programme can be implemented. For 2000 people to finish the CDSMP programme and have them evaluated at 6 months, between 100 and 140 workshops will have to be imparted, 20 to 28 per country. In practice, at each study site one workshop should be started every month during approximately one year and a half. A drop-out rate of minimum 20% is assumed.

Evaluation

The last phase of the project is its evaluation in terms of effectiveness of the programme to bring about changes in health-related behaviour, self-efficacy and quality of life. Furthermore, the economic and efficiency impact will be analyzed by means of cost-benefit analyses. An auto-administrable impact questionnaire has been designed to assess these different outcome measures at baseline (T0, before the CDSMP) and at 6 months (T1).

Sample size

The study primarily wishes to demonstrate a statistically significant difference on the two subscales of the 12 item short form SF-12[22]measuring Health-Related Quality of Life, and in the five participating countries. Based on a study on the effect of the application of the CDSMP in lumbar back pain subjects the sub-score “well-being” and “energy” of the SF-36 improved 6.0 +/- 19.1 and 4.3 +/- 23.2, respectively after six months.[23] The lowest Cohen’s d is therefore of 0.185 in quality of life domains. Thus, the principal conclusion of the study will rely on 10 tests. Applying the Bonferroni’s correction on the significance threshold, we will consider an alpha risk of 0.005 for the tests of the objective to increase the quality of life (self-rated with SF-12) in patients with chronic disease and caregivers in a socioeconomic-vulnerable condition. To show a statistically significant effect size of 0.185, with a bilateral alpha risk of 0.005, a power of 0.9, and a correlation between baseline and 6 months of 0, by a paired Student’s t test, we need to analyze 978 subjects. Assuming a drop-out rate of 20%, we need to include at least 1223 subjects. The multicentric study will include 2,500 subjects (500 per country), which will bring about sufficient power to analyze the objectives.

Statistical analysis plan

Differences between baseline and follow-up measurements will be assessed by means of the paired T test (for normal distributed variables), the Mann Whitney U test (for variables not normally distributed) or Pearson Chi-square test (for variable fractions). To adjust for multiple testing, one-way analyses of variance with post hoc testing (type Bonferroni) will be performed. The relationship of an outcome measure (T1) with explanatory variables will be determined using ordinary least squares (OLS) regression analysis. The outcome measure (T0), (change in) other outcome measures, risk factors and social determinants may serve as explanatory variables. In

For peer review only

addition, the above-mentioned analyses will be repeated for each country separately and possibly for other subgroups (variables which are likely to influence the effect of the intervention itself, e.g. age and sex). All data from each of the five pilot sites will be anonymized and centralized at one study site for statistical analysis. Analyses will be performed with SPSS version 25.0 (IBM SPSS Statistics for Windows, Armonk, NY: IBM Corp).

Guidelines and Policy recommendations

Based on the results of the project, the identification of the most commonly extended Best Practices in the field of Public Health and Chronis Diseases management in Europe and an analysis of country-specific contexts, guidelines and policy recommendations will be formulated to allow the scaling up of community-based interventions in vulnerable populations.

Patient and public involvement

Patients were not involved in the design of the study, choice of outcome measures or definition of the recruitment strategies. However, some patients and caregivers take an active part in the conduct of the study as trained monitors, facilitating workshop sessions in the community. Study participants will be able to access the study results on request as stated in the participant information letter. News on the study progress is being published via various general and specialized media channels throughout the running of the study.

For peer review only

Ethics and dissemination

It is not regarded as ethical to merely conduct descriptive research on vulnerable subjects and not run specific interventions afterwards. For this reason, no control group is included in the study, being consistent with the principle of social justice and the ethics of working with marginalized groups. [24]

The study follows the directives of the Helsinki declaration and the corresponding ethical regulations are being respected at each study site. In Occitanie, France, the Ethics Committee of the South-west and Oversees I in Toulouse (Comité de Protection des Personnes Sud-Ouest et Outre-Mer I) approved the study on 05/11/2018; study number 9788. In Genoa, Italy, the Regional Ethics Committee of Liguria (Il Comitato Etico della Regione Liguria) approved the study on 27/03/2018; study number 152-2018. In Rotterdam, The Netherlands, the Medical Ethics Review Committee (Medische Ethische Toetsings Commissie; METC) of the Erasmus MC University Medical Center - Rotterdam approved the study on 23/11/2017; study number MEC-2017-1116
In Asturias, Spain, the Research ethics committee of the Principality of Asturias (Comité de Ética de la Investigación del Principado de Asturias) approved the study on 31/01/2017; study number 20/17. In the United Kingdom, the online decision tool and query line of the Health Research Authority were consulted and it was concluded that approval from the NHS Research Ethics Committee was not necessary.

Eligible participants will be informed in person of the objectives and the procedures of the CDSMP by the investigator and in writing by the participant information letter prior to being enrolled. Only after a sufficient period of reflection and after clarification of all questions, the participant is asked to sign two copies of the informed consent form. Participation to the study is completely voluntary and participants have the right to withdraw their consent from the study at any time for any reason.

Data processing, communication and transfer are done in accordance with Regulation (EU) 2016/679 of the European Parliament and of the Council of 27 April 2016, also known as the GDPR. [25] Only the research team and the health authorities (if applicable), who have the duty to maintain confidentiality, will have access to all collected study data. Only information that cannot be identified may be transmitted to third parties. Information transmission to other countries will be carried out anonymously with a data protection level according to the EU Regulation 2016/679. The data will be collected and preserved until the study is finished in a codified way. The person responsible for data custody is responsible for the data file and treatment. At the end of the study, the data will be anonymized.

Via promotional materials, use of mass media, online activities, presentations at events and scientific publications, the project's activities, progress and outcomes will be disseminated as described in a specific communication and dissemination plan. Also, this strategy aims to enhance the engagement of health and social care providers, civil society and public administration officers, all of which considered multipliers due to their broad network and potential influence on the target population. Nevertheless, the main intended beneficiaries of the strategy are vulnerable

For peer review only

people with long term conditions and the health services serving these people. Accessing the hard-to-reach is one of the mayor challenges in EFFICHRONIC for which a solid strategy will be used.

For peer review only

Author contributions

MMPG designed and specified the initial study protocol. ALDB, VV, SST, YMP, RVA, DPM, GB, AP, SZ, HR and JRH advised on the study protocol. ALDB and MMPG drafted the manuscript. All authors critically revised the manuscript form important content and approved the final manuscript.

Funding statement

This work was supported by the European Union's Health Programme (2014-2020) grant number 738127.

Competing interests statement

The authors have no competing interests to declare.

Acknowledgements

The members of the EFFICHRONIC Consortium are: Marta M Pisano Gonzalez, Raquel Vazquez Alvarez, Delia Peñacoba Maestre (Health Service of the Principality of Asturias – SESPA, Spain); Inés Rey Hidalgo and Raquel Ochoa Gonzalez (the Foundation for the Promotion in Asturias of applied Scientific Research and Technology – FICYT, Spain); An LD Boone and José Ramón Hevia Fernandez (Public Health General Directorate, Principality of Asturias – CSPA, Spain); Yves-Marie Pers, Christian Jorgensen, Verushka Valsecchi, Rosanna Ferreira, Adrien Durand, Cristina Balaguer, Céline Cardoso and Manuel Ramos (The University Hospital Center of Montpellier – CHUM, France); Graham Baker, Danni Brown and Suzanne Lucas (Qismet, Portsmouth, UK); Siok-Swan Tan, Irene Fierloos, Xuxi Zhang, Petra de Vries, Hein Raat (Erasmus MC University Medical Center, Rotterdam, The Netherlands); Alberto Pilotto, Sabrina Zora, Alberto Ferri, Alberto Cella and Alessandra Argusti (EO Galliera Hospital, Genua, Italy); Concepción Doñate Martínez and Jorge Garcés (University of Valencia, Polibienestar Research Institute – UVEG, Valencia, Spain).

For peer review only

REFERENCES

[1] Barnett K, Mercer S, Norbury,M, *et al*. Epidemiology of multimorbidity and implications for health care, research, and medical education: a cross-sectional study. *The Lancet* 2012; 380(9836): 37-43.

[2] World Health Organization [Internet]. Geneva; c2019 [cited 2019 Feb 13] [1 screen]. Available from: https://www.who.int/emergencies/ten-threats-to-global-health-in-2019.

[3] World Health Organization [Internet]. Geneva; c2018 [cited 2019 Feb 13] [pdf file]. Available from: http://apps.who.int/gb/ebwha/pdf_files/WHA71/A71_4-en.pdf.

[4] Muka T, Imo D, Jaspers L, *et al*. The global impact of non-communicable diseases on healthcare spending and national income: a systematic review. *Eur J Epidemiol* 2015; 30:251–277.

[5] Dalstra JA, Kunst AE, Borrell C, *et al*. Socioeconomic differences in the prevalence of common chronic diseases: an overview of eight European countries. *International journal of epidemiology 2005; 34*(2): 316-326.

[6] Kontis V, Mathers CD; RehmJ, *et al*. Contribution of six risk factors to achieving the 25× 25 non-communicable disease mortality reduction target: a modelling study. *The Lancet* 2014; *384*(9941): 427-437.

[7] Marmot M, Wilkinson R. *Social determinants of health*. Oxford: Oxford University Press, 2006.

[8] Hart J. The inverse care law. *The Lancet* 1971*; 297*(7696): 405-412.

[9] Bauer UE, Briss PA, Goodman RA, *et al*. Prevention of chronic disease in the 21st century: elimination of the leading preventable causes of premature death and disability in the USA. *The Lancet* 2014*;* 384(9937): 45-52.

[10] Bodenheimer T, Lorig K, HolmanH, *et al*. Patient self-management of chronic disease in primary care. *Jama* 2002*; 288*(19): 2469-2475.

[11] Smith ML, Wilson MG, Robertson MM, *et al*. Impact of a Translated Disease Self-Management Program on Employee Health and Productivity: Six-Month Findings from a Randomized Controlled Trial**.** *Int J Environ Res Public Health* 2018; 15(5): 851.

[12] Barr VJ, Robinson S, Marin-Link B *et al*. The expanded Chronic Care Model: an integration of concepts and strategies from population health promotion and the Chronic Care Model. *Healthcare Quarterly* 2003; *7*(1).

[13] Lorig K, Sobel DS, Ritter PL, *et al*. Effect of a self-management program on patients with chronic disease. *Eff. Clin. Pract*. 2001;4:256–262.

[14] Bandura A. Social cognitive theory of self-regulation. *Organ. Behav. Hum. Decis. Process* 1991;50:248–287.

[15] Lorig K, Sobel DS, Stewart AA, *et al*. Evidence suggesting that a chronic disease self-management program can improve health status while reducing hospitalization: A randomized trial. *Med. Care.* 1999;37:5–14.

[16] Ory MG, Smith ML, Kulinski KP, *et al*. Self-management at the tipping point: Reaching 100,000 Americans with evidence-based programs. *J. Am. Geriatr. Soc*. 2013;61:821–823.

[17] Harrell JS, Bangdiwala SL, Deng S. Smoking initiation in youth: the roles of gender, race, socioeconomics, and developmental status. *Journal of Adolescent Health* 1998; *23*(5): 271-279.

[18] Porter SN. Poverty, Discrimination, and Health. *Social Pathways to Health Vulnerability* 2019; 23-53.

[19] Malgesini RG, Rua VA, Martinez IZ. Informe sobre la Vulnerabilidad Social. *Cruz Roja Española* 2015; 1-120.

[20] Pilotto A, Ferrucci L, Franceschi M, *et al*. Development and validation of a multidimensional prognostic index for one-year mortality from comprehensive geriatric assessment in hospitalized older patients. *Rejuvenation research* 2018; *11*(1): 151-161.

[21] Pilotto A, Veronese N, Quispe K, *et al*. Development and validation of a self-administered Multidimensional Prognostic Index (SELFY-MPI) to predict negative health outcomes in community-dwelling persons. *Rejuvenation research* 2018.

[22] Ware J, Kosinski M, Keller SD. A 12-Item Short-Form Health Survey: construction of scales and preliminary tests of reliability and validity. Med Care 1996; 34(3):220-233.

[23] Haas M, Groupp E, Muench J, et al. Chronic Disease Self-Management Program for Low Back Pain in the Elderly. Journal of Manipulative and Physiological Therapeutics 2005; 28 (4): 228-237.

[24] Mosavel M, Simon C. Exploratory Health Disparities Research: The Need to Provide a Tangible Benefit to Vulnerable Respondents. Ethics & Behavior 2010; 20(1), 1–9.

[25] https://eur-lex.europa.eu/legal-content/EN/TXT/?uri=celex%3A32016R0679 (accessed 13 Feb 2019).

For peer review only

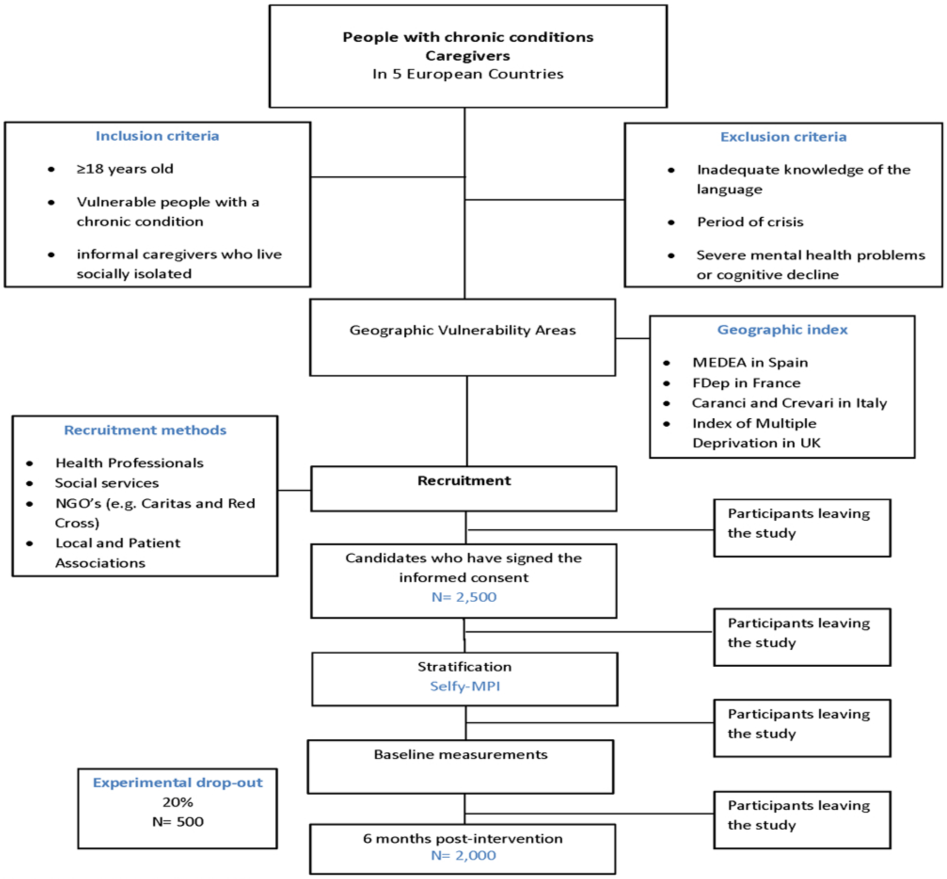

Figure 1: Flow diagram of EFFICHRONIC

90x90mm (300 x 300 DPI)

BMJ Open

EFFICHRONIC: a non-controlled, multicentre European prospective study to measure the efficiency of a Chronic Disease Self-Management Programme in socio-economic vulnerable population.

Journal:	*BMJ Open*
Manuscript ID	bmjopen-2019-032073.R1
Article Type:	Protocol
Date Submitted by the Author:	27-Sep-2019
Complete List of Authors:	Boone, An LD; Gobierno del Principado de Asturias Consejeria de Sanidad<br>Pisano-Gonzalez, Marta ; Health Service of the Principality of Asturias<br>Valsecchi, Verushka; Centre Hospitalier Universitaire de Montpellier<br>Tan, Siok; Erasmus Medical Center<br>Pers, Yves-Marie; Centre Hospitalier Universitaire de Montpellier<br>Vazquez-Alvarez, Raquel; Health Service of the Principality of Asturias<br>Peñacoba-Maestre, Delia; Health Service of the Principality of Asturias<br>Baker, Graham; QISMET<br>Pilotto, Alberto; Ente Ospedaliero Ospedali Galliera<br>Zora, Sabrina; Ente Ospedaliero Ospedali Galliera<br>Raat, Hein; Erasmus Medical Center<br>Hevia-Fernandez, Jose Ramón; Gobierno del Principado de Asturias Consejeria de Sanidad
**Primary Subject Heading**:	Public health
Secondary Subject Heading:	Evidence based practice, Health economics, Health policy, Patient-centred medicine
Keywords:	Chronic Disease, Social Determinants of Health, Caregivers, Patient participation, Self-Management, Cost-efficiency

SCHOLARONE™
Manuscripts

TITLE PAGE

Title

EFFICHRONIC: a non-controlled, multicentre European prospective study to measure the efficiency of a Chronic Disease Self-Management Programme in socio-economic vulnerable population.

Authors

An LD Boone (corresponding author), Government of the Principality of Asturias, Ministry of Health, C/ Ciriaco Miguel Vigil, 9, 33006 Oviedo – Asturias, Spain. Member of the research group 'Atención comunitaria y envejecimiento activo´, Inst Invest Sanitaria Principado Asturias ISPA, Oviedo, Spain, an.boone@asturias.org, phone: +34 985 10 63 23, ext. 17638, fax: +34 985 10 63 20.

Marta M Pisano-González, SESPA, Health Service of the Principality of Asturias; Oviedo, Spain. Member of the research group 'Atención comunitaria y envejecimiento activo´, Inst Invest Sanitaria Principado Asturias ISPA, Oviedo, Spain.

Verushka Valsecchi, Unité d'Immunologie Clinique et Thérapeutique ostéo-articulaire, Centre Hospitalier Universitaire (CHU) de Montpellier; Montpellier, France.

Siok-Swan Tan, Department of Public Health, Erasmus MC University Medical Center; Rotterdam, the Netherlands.

Yves-Marie Pers, Unité d'Immunologie Clinique et Thérapeutique ostéo-articulaire, Centre Hospitalier Universitaire (CHU) de Montpellier; Montpellier, France.

Raquel Vazquez-Alvarez, SESPA, Health Service of the Principality of Asturias; Oviedo, Spain.

Delia Peñacoba-Maestre, SESPA, Health Service of the Principality of Asturias; Oviedo, Spain.

Graham Baker, Quality Institute for Self Management Education & Training (QISMET); Portsmouth, United Kingdom.

Alberto Pilotto, Department of Geriatric Care, E.O. Galliera Hospital; Genova, Italy.

Sabrina Zora, Department of Geriatric Care, E.O. Galliera Hospital; Genova, Italy.

Hein Raat, Department of Public Health, Erasmus MC University Medical Center; Rotterdam, the Netherlands.

Jose Ramón Hevia-Fernandez, Government of the Principality of Asturias, Ministry of Health; Oviedo, Spain.

On behalf of the EFFICHRONIC Consortium

For peer review only

Word count: 3980

Keywords: Chronic Disease, Social determinants of Health, Caregivers, Patient participation, Self-Management

For peer review only

Abstract

INTRODUCTION

More than 70% of mortality worldwide is due to chronic conditions. In addition, it has been proven that social determinants have an enormous impact on health-related behaviour and on the received attention from healthcare services, producing health inequalities. The objective of this study is to reduce the burden of chronic diseases in five European regions, focusing on vulnerable populations, and to increase the sustainability of health systems by implementing a chronic disease self-management programme (CDSMP).

METHODS AND ANALYSIS

2,000 people with chronic conditions or informal caregivers belonging to vulnerable populations will be enrolled in the CDSMP in Spain, Italy, United Kingdom, France and the Netherlands. Inclusion of patients will be based on a geographical, socio-economic and clinical stratification process. The programme will be evaluated on self-efficacy, quality of life and cost-effectiveness by means of a combination of validated questionnaires at baseline and at 6 months from baseline.

ETHICS AND DISSEMINATION

This study will follow the directives of the Helsinki Declaration and will adhere to the European Union (EU) General Data Protection Regulation (GDPR). The project's activities, progress and outcomes will be disseminated via promotional materials, use of mass media, online activities, presentations at events and scientific publications.

REGISTRATION DETAILS

The trial was registered at ISRCTN on 20/06/2018 with registry number 70517103.

For peer review only

Article Summary

Strengths and limitations of this study

- This study will gather valuable data on the CDSMP implementation in five European countries with different cultures, socio-economic realities, health systems and regulations.
- Particularly, the used recruitment strategies will provide important information on how to enroll vulnerable people, who have the greatest potential for reducing the burden of chronic diseases.
- Furthermore, the study will provide scientific data on the reliability of investment in chronic disease prevention.
- The study is not randomized controlled to allow all possible beneficiaries to participate although unfortunately, vulnerable people who do not speak the local language cannot be included.
- It has been demonstrated that the positive results of these kinds of interventions reach their highest impact at one year post-intervention so ideally, a longer-term trial should be conducted.

For peer review only

Introduction

In the 21st century, chronic conditions are among the most important public health challenges mainly due to their double impact: the human suffering they cause on the one hand and the damage they produce to global socio-economic development on the other. Despite the fact that healthcare systems are still primarily focusing on acute diseases[1], they are slowly transforming because of demographic changes, the increasing prevalence of chronic diseases, and the budgetary crisis of recent years. This shift requires new healthcare delivery models to ensure sustainability.

In fact, chronic conditions and also vulnerable settings[2 3] are part of the ten threats to global health the World Health Organization (WHO) has warned of recently. More than 70% of mortality worldwide (41 million people each year), is due to chronic conditions, such as cancer, diabetes, respiratory disorders or heart diseases. All of them are related to five main risk factors: tobacco use, physical inactivity, harmful consumption of alcohol, unhealthy diet and air pollution. Furthermore, 22% of the global population lives in vulnerable settings which exist in almost all regions of the world. As a result, the 13th WHO General Program of Work (2019 – 2023), expresses this particular concern with the slogan: "Promote health, keep the world safe, serve the vulnerable", urging precisely to work on health promotion in populations suffering particular vulnerability.[3]

The large impact of Non-Communicable Diseases (NCD) on national income is primarily due to loss of productivity by cause of absenteeism and inability to work, threatening the sustainability of recent macroeconomic achievements. Furthermore, there has been a steady global increase in healthcare expenditure on NCDs over the years. According to a systematic review on healthcare spending and national income, cardiovascular diseases account for 12% of healthcare expenditure in the European Union and diabetes related costs were estimated at 7%.[4] Besides, other conditions and risks factors such as depression or obesity are becoming more prevalent in higher-income EU countries and are greatly determined by poverty and low-income. [5 6]

In addition, recent scientific evidence has shown the enormous impact of social determinants (i.e. income, unemployment, housing quality, educational level or gender) on health, manifested in diverse short- and long-term health inequalities.[7] These differences are not only related to deficient self-care behaviour[1] and poor health, but also to the received attention from healthcare services. Vulnerable people are generally less likely to receive preventive care, timely diagnosis, or follow-up care for their chronic conditions. These observations agree with the 'inverse care law' theory described in 1971[8] by JT Hart, referring to the fact that often, the availability of good medical or social care varies inversely with the need of the population served. This way, chronic conditions and deprivation are associated in a vicious circle.

Fortunately, and as evidence shows, chronic conditions can be controlled by reducing their risk factors[9], which are mostly linked to unhealthy lifestyles, and health literacy programmes that focus on self-efficacy and empowerment can successfully modify many harmful habits. [10 11]

For peer review only

The basis of the EFFICHRONIC project is the internationally acknowledged Chronic Care Model (CCM)[12] which aims to promote patients' empowerment and autonomy. The specific programme used in the project is the Chronic Disease Self-Management Programme (CDSMP).[13] It concerns a process-centered intervention constructed on the Social Learning Theory[14] and it is appropriate for adults living with one or more chronic conditions. Since its beginning in 1990[15], the effectiveness of the CDSMP has been demonstrated by means of positive health outcomes and more efficient use of health resources.[16] The programme has a community focus and is disseminated through an international infrastructure of training, certification and licensing.

Study Rationale

Up to date, prevention research still focuses mainly on disease specific interventions and few studies on particular vulnerable populations are performed. Furthermore, data on the efficiency and cost-benefit of prevention programmes is scarce, which reduces policy engagement and likewise, it prevents health systems from being more sustainable and efficient.[17 18]

EFFICHRONIC aims to empower individuals from (socially) deprived communities to manage their chronic conditions, in an integrated and non-disease specific way. The overall objective of the project is to reduce the burden of chronic conditions and increase the sustainability of health systems by implementing a chronic disease self-management programme in socio-economic vulnerable populations in five European regions.

Five specific objectives have been defined: 1) to identify vulnerable people by means of a multidimensional analysis in the involved regions; 2) to design specific recruitment strategies to reach the target population; 3) to implement the programme in the five regions with at least 400 individuals per region; 4) to generate a comprehensive impact assessment framework, including cost-efficiency and health-economic assessment; and 5) to define guidelines and policy recommendations to allow to the scaling up of the EFFICHRONIC methodology to other regions in Europe.

For peer review only

Methods and analysis

Methodology

The methodology used for the community intervention of EFFICHRONIC is the “Chronic Disease Self-Management Programme" developed at Stanford University. The CDSMP is a community based public health intervention provided by trained monitors. The programme is strongly evidence-based and consists of the following conceptual elements: self-efficacy, empowerment, salutogenesis, peer-to-peer education and vicarious training. It includes action planning and feedback about problem-solving skills and other competences such as the reinterpretation of symptoms and training in disease management.

A cascade training model is used in the CDSMP method. On top are Self-Management Resource Centre (SMRC) licensed T-trainers who possess the maximum teaching expertise, in charge of training Master Trainers, who in turn train monitors (or leaders) who will conduct the chronic disease self-management workshops. The monitors receive practical training on the philosophical and methodological basis of the CDSMP: empowerment, self-efficacy, group management, management of painful emotions, positive health, and efficient communication among other skills. Once trained, the monitors facilitate the workshops in pairs. In each country minimum 32 monitors will be trained to instruct the end-users: people with chronic conditions or caregivers. The CDSMP workshops have a duration of 6 weeks with sessions of 2,5 hours once a week in groups of 12 to 20 participants. The programme fidelity and quality plan include the supervision of workshops in the community by the Master Trainers by means of periodic protocolled monitor audits. Workshop assistants receive a handout with the workshop topics and homework assignments, a consultation book and a certificate of attendance if they were present at minimum 4 of the 6 sessions.

Study design

EFFICHRONIC is a multicentre non-controlled prospective study with a duration of three years in five European settings: the Principality of Asturias in Spain, the area of Genoa in Italy, several regions in the United Kingdom, the Occitanie region in France and the area of Rotterdam in the Netherlands. The participating countries represent a wide range of social, economic and healthcare-related realities. The EFFICHRONIC intervention consists of the following phases: identification, recruitment and stratification of participants from vulnerable populations, implementation of the intervention and an economic and efficiency evaluation with a pre-post design. Additionally, guidelines and policy recommendations will be developed based on the project conclusions to allow the scaling-up of the intervention to other contexts.

Study population

The final study population will consist of 2,000 vulnerable participants distributed over the five implicated areas, who finish the CDSMP (4 out of 6 sessions) and who are evaluated 6 months

For peer review only

after the intervention. Taking into account a drop-out rate of minimum 20%, at least 2,500 participants will be recruited initially.

The vulnerability concept of EFFICHRONIC is based on a definition from the Spanish Red Cross which describes it as “a situation between complete social inclusion and total exclusion”.[19] Keeping in mind this definition and adapting it to some specific requirements of the CDSMP (e.g. being able to understand the local language) and of EFFICHRONIC (being traceable for follow-up at six months), the inclusion criteria were defined (Figure 1).

The target population involves two main groups: vulnerable people with at least one chronic condition, and informal caregivers who live socially isolated. In general, participants must be minimum 18 years old, reside in the selected geographical areas, have their basic housing needs met, and possess an adequate knowledge of the local language.

Participants with chronic conditions are either older than 65 living alone or in a nursing home; have difficulties to make ends meet (especially if belonging to an ethnic minority or being a legal immigrant, asylum seeker or refugee); or are imprisoned. A chronic disease, self-reported or clinically evaluated, is outlined as a pathology listed in the International Classification of Primary Care (ICPC-2) with a code between 70 and 99 in one of the 17 chapters, and should have more than six months of evolution.

Informal caregivers are defined as persons looking after somebody ill, usually a family member, without receiving payment. Additionally, to participate in the study, it is required that they are socially isolated, as is the case of carers living in remote areas without transportation or with limited access to it (without a private car and public transport at more than one kilometre from the house), without internet access or little social support.

General exclusion criteria are: going through a period of crisis (e g being evicted), having severe mental health problems that cause distorted perception of reality and/or inability to participate in group dynamics, having cognitive decline (e.g. Alzheimer´s) or having active addictive disorders. Although the excluded groups are among the most vulnerable of society, they need other and more specific interventions prior to be eligible for a self-management programme.

Sample size

The study primarily wishes to demonstrate a statistically significant difference on the two subscales of the 12 item Short Form (SF-12)[20] measuring Health-Related Quality of Life, and in the five participating countries. Based on a study on the effect of the CDSMP in lumbar back pain, the subscores “well-being” and “energy” of the SF-36 improved 6.0 +/- 19.1 and 4.3 +/- 23.2, respectively after six months.[21] The lowest Cohen’s d is therefore of 0.185 in quality of life domains. Thus, the principal conclusion of the study will rely on 10 tests. Applying the Bonferroni’s correction on the significance threshold, we will consider an alpha risk of 0.005 for the tests measuring quality of life (self-rated with SF-12) in vulnerable patients with chronic diseases and caregivers. To show a statistically significant effect size of 0.185, by a paired Student’s T test, with

For peer review only - http://bmjopen.bmj.com/site/about/guidelines.xhtml

a bilateral alpha risk of 0.005, a power of 0.9, and a correlation between baseline and 6 months of 0, we need to analyze 978 subjects. Assuming a drop-out rate of 20%, we need to include at least 1223 subjects. The multicentre study will include 2,000 subjects (400 per country), which will bring about sufficient power to analyze the objectives.

Sample selection and recruitment

The potential participants are being identified by means of a geographical, socio-economic and clinical stratification process. A distinction is made between the individual and social dimension of vulnerability, leading to different recruitment strategies that will be combined to be more efficient. First, geographical mapping of areas with a higher percentage of vulnerable population was done based on existing national and local indices in all participating countries. Consecutively, individualized recruitment will take place in these areas by means of specifically designed actions. Multiple recruitment channels are used in coordination with local agents from the healthcare system, social services and community-based structures. More specifically, the following profiles and organizations are involved in recruitment: health professionals, pharmacists, social workers, Non-Governmental Organisations (NGOs), city councils and associations. The recruitment strategy at each study site is adapted to the specific local circumstances and collaborating organizations.

Stratification

To identify the people who will benefit most from the programme, individual socio-economic and clinical parameters will be measured by a multidimensional stratification analysis. The tool used for this purpose, the "SELFY-MPI", is developed based on the validated Multidimensional Prognostic Index (MPI).[22] The new tool is self-administrable and was specifically designed for EFFICHRONIC,[23] adapting it to the broad adult population (not limited to the elderly), and incorporating the socio-economic parameters from the Spanish Gijón scale: education, income, co-habitation, age, and social support. [24] Income categories are country specific and built on the corresponding minimum wages.

Implementation

Once the target population is identified and recruitment is ongoing, the programme can be implemented. In order to reach the target sample size of 2,000 people, between 100 and 140 workshops (20 to 28 per country) will have to be conducted. In practice, one workshop will start every month during approximately one year and a half at each study site.

Evaluation

The last phase of the project consists of evaluating the effectiveness of the programme to change health-related behaviour, self-efficacy and quality of life. Furthermore, the economic and efficiency impact will be analyzed by means of cost-benefit analyses. An auto-administrable impact questionnaire, containing validated scales and measuring tools, has been designed to assess these different outcome measures at baseline (T0, before the CDSMP) and at 6 months (T1).[25]

For peer review only

Statistical analysis plan

Differences between baseline and follow-up measurements will be assessed by means of the paired T test (for normal distributed variables), the Mann Whitney U test (for variables not normally distributed) and the Pearson Chi-square test (for variable fractions). To adjust for multiple testing, one-way analyses of variance with post hoc testing (type Bonferroni) will be performed. The relationship of an outcome measure (T1) with explanatory variables will be determined using ordinary least squares (OLS) regression analysis. The outcome measure (T0), (change in) other outcome measures, risk factors and social determinants may serve as explanatory variables. In addition, the above-mentioned analyses will be repeated for each country separately and possibly for other subgroups (for variables which are likely to influence the effect of the intervention itself, e.g. age and sex). All data from each of the five pilot sites will be anonymized and centralized at one study site for statistical analysis. Analyses will be performed with SPSS version 25.0 (IBM SPSS Statistics for Windows, Armonk, NY: IBM Corp).

Guidelines and Policy recommendations

By the end of the project, guidelines and policy recommendations will be formulated based on the specific results of the participating countries and taking into account already known Best Practices at European level, to allow the scaling up of community-based interventions in vulnerable populations.

Patient and public involvement

Patients were not involved in the design of the study, choice of outcome measures or definition of the recruitment strategies. However, some patients and caregivers take an active part in the conduct of the study as trained monitors, facilitating workshop sessions in the community. Study participants will be able to access the study results on request as stated in the participant information letter. News on the study progress is being published via various general and specialized media channels throughout the running of the study.

For peer review only

Ethics and dissemination

It is not considered ethical to conduct descriptive research on vulnerable subjects without running specific interventions afterwards. For this reason, the study is not randomised-controlled, being consistent with the principle of social justice and the ethics of working with marginalized groups. [26]

The study follows the directives of the Helsinki declaration and the corresponding ethical regulations are being respected at each study site. In Occitanie, France, the Ethics Committee of the South-west and Oversees I in Toulouse (Comité de Protection des Personnes Sud-Ouest et Outre-Mer I) approved the study on 05/11/2018; study number 9788. In Genoa, Italy, the Regional Ethics Committee of Liguria (Il Comitato Etico della Regione Liguria) approved the study on 27/03/2018; study number 152-2018. In Rotterdam, the Netherlands, the Medical Ethics Review Committee (Medische Ethische Toetsings Commissie; METC) of the Erasmus MC University Medical Center - Rotterdam approved the study on 23/11/2017; study number MEC-2017-1116. In Asturias, Spain, the Research Ethics Committee of the Principality of Asturias (Comité de Ética de la Investigación del Principado de Asturias) approved the study on 31/01/2017; study number 20/17. In the United Kingdom, the online decision tool and query line of the Health Research Authority were consulted and it was concluded that approval from the NHS Research Ethics Committee was not necessary.

Eligible participants will be informed of the objectives and the procedures of the CDSMP in person by the investigator and in writing by a participant information letter prior to enrolment. Only after a sufficient period of reflection and after clarification of all questions, the participant is asked to sign two copies of the informed consent form. Participation to the study is completely voluntary and participants have the right to withdraw their consent from the study at any time for any reason.

Data processing, communication and transfer are done in accordance with EU Regulation 2016/679 of the European Parliament and of the Council of 27 April 2016, also known as the GDPR. [27] Only the research team that has the duty to maintain confidentiality has access to all collected study data. Only information that cannot be identified may be transmitted to third parties. Information transmission to other countries will be carried out anonymously with a data protection level according to the mentioned regulation. The data will be collected and preserved until the study is finished in a codified way. The person responsible for data custody is responsible for the data file and treatment. At the end of the study, the data will be anonymized.

Via promotional materials, use of mass media, online activities, presentations at events and scientific publications, the project's activities, progress and outcomes will be disseminated as described in a specific communication and dissemination plan. The strategy aims to enhance the engagement of healthcare and social care providers, civil society and public administration officers, all of which multipliers due to their broad network and potential influence on the target population. Nevertheless, the main intended beneficiaries of the strategy are vulnerable people

For peer review only - http://bmjopen.bmj.com/site/about/guidelines.xhtml

with long-term conditions and the health services serving these people. Accessing the hard-to-reach is one of the mayor challenges in EFFICHRONIC for which a solid strategy will be used.

For peer review only

Author contributions

MMPG designed the initial study protocol. ALDB, VV, SST, YMP, RVA, DPM, GB, AP, SZ, HR and JRHF advised on the study protocol. ALDB and MMPG drafted the manuscript. All authors critically revised the manuscript on important content and approved the final manuscript.

Funding statement

This work was supported by the European Union's Health Programme (2014-2020) grant number 738127.

Competing interests statement

The authors have no competing interests to declare.

Acknowledgements

The members of the EFFICHRONIC Consortium are: Marta M Pisano Gonzalez, Raquel Vazquez Alvarez, Delia Peñacoba Maestre (Health Service of the Principality of Asturias – SESPA, Spain); An LD Boone and José Ramón Hevia Fernandez (Public Health General Directorate, Principality of Asturias – CSPA, Spain); Sonia López-Villar, Inés Rey Hidalgo and Raquel Ochoa Gonzalez (the Foundation for the Promotion in Asturias of applied Scientific Research and Technology – FICYT, Spain); Yves-Marie Pers, Christian Jorgensen, Verushka Valsecchi, Rosanna Ferreira, Adrien Durand, Cristina Balaguer, Céline Cardoso and Manuel Ramos (The University Hospital Center of Montpellier – CHUM, France); Graham Baker, Danni Brown and Suzanne Lucas (Qismet, Portsmouth, UK); Siok-Swan Tan, Irene Fierloos, Xuxi Zhang, Petra de Vries, Hein Raat (Erasmus MC University Medical Center, Rotterdam, The Netherlands); Alberto Pilotto, Sabrina Zora, Alberto Ferri, Alberto Cella and Alessandra Argusti (EO Galliera Hospital, Genua, Italy); Concepción Doñate Martínez and Jorge Garcés (University of Valencia, Polibienestar Research Institute – UVEG, Valencia, Spain).

Figure legend

Figure 1: EFFICHRONIC: inclusion criteria flow chart

For peer review only

REFERENCES

[1] Barnett K, Mercer S, Norbury M, *et al*. Epidemiology of multimorbidity and implications for health care, research, and medical education: a cross-sectional study. *The Lancet* 2012; 380(9836): 37-43.
[2] World Health Organization [Internet]. Geneva; c2019 [cited 2019 Feb 13] [1 screen]. Available from: https://www.who.int/emergencies/ten-threats-to-global-health-in-2019.
[3] World Health Organization [Internet]. Geneva; c2018 [cited 2019 Feb 13] [pdf file]. Available from: http://apps.who.int/gb/ebwha/pdf_files/WHA71/A71_4-en.pdf.
[4] Muka T, Imo D, Jaspers L, *et al*. The global impact of non-communicable diseases on healthcare spending and national income: a systematic review. *Eur J Epidemiol* 2015; 30:251–277.
[5] Dalstra JA, Kunst AE, Borrell C, *et al*. Socioeconomic differences in the prevalence of common chronic diseases: an overview of eight European countries. *International journal of epidemiology 2005; 34*(2): 316-326.
[6] Kontis V, Mathers CD; Rehm J, *et al*. Contribution of six risk factors to achieving the 25× 25 non-communicable disease mortality reduction target: a modelling study. *The Lancet* 2014; *384*(9941): 427-437.
[7] Marmot M, Wilkinson R. Social determinants of health. Oxford: *Oxford University Press*, 2006.
[8] Hart J. The inverse care law. *The Lancet* 1971*; 297*(7696): 405-412.
[9] Bauer UE, Briss PA, Goodman RA, *et al*. Prevention of chronic disease in the 21st century: elimination of the leading preventable causes of premature death and disability in the USA. *The Lancet* 2014*;* 384(9937): 45-52.
[10] Bodenheimer T, Lorig K, Holman H, *et al*. Patient self-management of chronic disease in primary care. *Jama* 2002*; 288*(19): 2469-2475.
[11] Smith ML, Wilson MG, Robertson MM, *et al*. Impact of a Translated Disease Self-Management Program on Employee Health and Productivity: Six-Month Findings from a Randomized Controlled Trial**.** *Int J Environ Res Public Health* 2018; 15(5): 851.
[12] Barr VJ, Robinson S, Marin-Link B *et al*. The expanded Chronic Care Model: an integration of concepts and strategies from population health promotion and the Chronic Care Model. *Healthcare Quarterly* 2003; *7*(1).
[13] Lorig K, Sobel DS, Ritter PL, *et al*. Effect of a self-management program on patients with chronic disease. *Eff. Clin. Pract*. 2001;4:256–262.
[14] Bandura A. Social cognitive theory of self-regulation. *Organ. Behav. Hum. Decis. Process* 1991; 50: 248–287.
[15] Lorig K, Sobel DS, Stewart AA, *et al*. Evidence suggesting that a chronic disease self-management program can improve health status while reducing hospitalization: A randomized trial. *Med. Care.* 1999;37:5–14.
[16] Ory MG, Smith ML, Kulinski KP, *et al*. Self-management at the tipping point: Reaching 100,000 Americans with evidence-based programs. *J. Am. Geriatr. Soc*. 2013;61:821–823.
[17] Harrell JS, Bangdiwala SL, Deng S. Smoking initiation in youth: the roles of gender, race, socioeconomics, and developmental status. *Journal of Adolescent Health* 1998; *23*(5): 271-279.
[18] Porter SN. Poverty, Discrimination, and Health. *Social Pathways to Health Vulnerability* 2019; 23-53.
[19] Malgesini RG, Rua VA, Martinez IZ. Informe sobre la Vulnerabilidad Social. *Cruz Roja Española* 2015; 1-120.
[20] Ware J, Kosinski M, Keller SD. A 12-Item Short-Form Health Survey: construction of scales and preliminary tests of reliability and validity. *Med Care* 1996; 34(3):220-233.

[21] Haas M, Groupp E, Muench J, *et al*. Chronic Disease Self-Management Program for Low Back Pain in the Elderly. *Journal of Manipulative and Physiological Therapeutics* 2005; 28 (4): 228-237.

[22] Pilotto A, Ferrucci L, Franceschi M, *et al*. Development and validation of a multidimensional prognostic index for one-year mortality from comprehensive geriatric assessment in hospitalized older patients. *Rejuvenation research* 2018; *11*(1): 151-161.

[23] Pilotto A, Veronese N, Quispe K, *et al*. Development and validation of a self-administered Multidimensional Prognostic Index (SELFY-MPI) to predict negative health outcomes in community-dwelling persons. *Rejuvenation research* 2019; 22(4): 299-305.

[24] Garcia-Caselles P, Miralles R, Arellano M ,*et al.* Validation of a modified version of the Gijon's social-familial evaluation scale (SFES): The "Barcelona SFES Version", for patients with cognitive impairment. *Arch. Gerontol. Geriatr. Suppl.* 2004; 9: 201–206.

[25] Tan SS, Pisano MM; Boone AL, et al. Evaluation Design of EFFICHRONIC: The Chronic Disease Self-Management Programme (CDSMP) Intervention for Citizens with a Low Socioeconomic Position. Int. J. Environ. Res. Public Health 2019; 16 (11): 1883.

[26] Mosavel M, Simon C. Exploratory Health Disparities Research: The Need to Provide a Tangible Benefit to Vulnerable Respondents. *Ethics & Behavior* 2010; 20(1), 1–9.

[27] European Union Law [internet] [cited 2019 Feb 13] [1 screen]. Available from https://eur-lex.europa.eu/eli/reg/2016/679/oj.

For peer review only

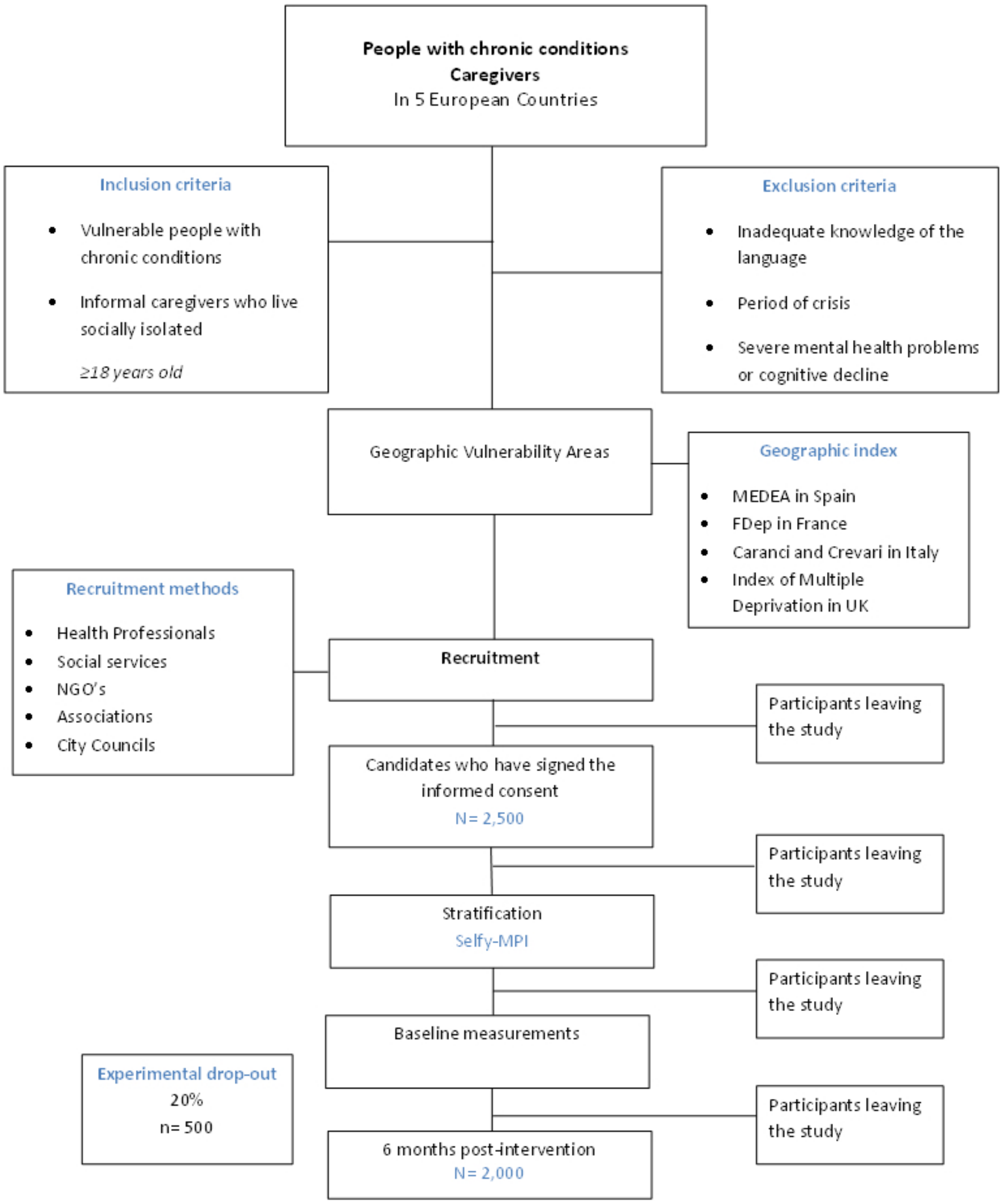

EFFICHRONIC: inclusion criteria flow chart

64x77mm (300 x 300 DPI)

BMJ Open

EFFICHRONIC study protocol: a non-controlled, multicentre European prospective study to measure the efficiency of a Chronic Disease Self-Management Programme in socio-economically vulnerable populations.

Journal:	*BMJ Open*
Manuscript ID	bmjopen-2019-032073.R2
Article Type:	Protocol
Date Submitted by the Author:	06-Nov-2019
Complete List of Authors:	Boone, An LD; Ministry of Health of the Principality of Asturias, Public Health General Directorate<br>Pisano-Gonzalez, Marta ; Health Service of the Principality of Asturias<br>Valsecchi, Verushka; Centre Hospitalier Universitaire de Montpellier<br>Tan, Siok; Erasmus Medical Center<br>Pers, Yves-Marie; Centre Hospitalier Universitaire de Montpellier<br>Vazquez-Alvarez, Raquel; Health Service of the Principality of Asturias<br>Peñacoba-Maestre, Delia; Health Service of the Principality of Asturias<br>Baker, Graham; QISMET<br>Pilotto, Alberto; Ente Ospedaliero Ospedali Galliera<br>Zora, Sabrina; Ente Ospedaliero Ospedali Galliera<br>Raat, Hein; Erasmus Medical Center<br>Hevia-Fernandez, Jose Ramón; Ministry of Health of the Principality of Asturias, Public Health General Directorate
**Primary Subject Heading**:	Public health
Secondary Subject Heading:	Evidence based practice, Health economics, Health policy, Patient-centred medicine
Keywords:	Chronic Disease, Social Determinants of Health, Caregivers, Patient participation, Self-Management, Cost-efficiency

SCHOLARONE™
Manuscripts

TITLE PAGE

Title

EFFICHRONIC study protocol: a non-controlled, multicentre European prospective study to measure the efficiency of a Chronic Disease Self-Management Programme in socio-economically vulnerable populations.

Authors

An L. D. Boone (corresponding author), Public Health General Directorate, Ministry of Health of the Principality of Asturias (CSPA), C/ Ciriaco Miguel Vigil, 9, 33006 Oviedo – Asturias, Spain. Member of the research group 'Atención comunitaria y envejecimiento activo´, Inst Invest Sanitaria Principado Asturias ISPA, Oviedo, Spain, an.boone@asturias.org, phone: +34 985 10 63 23, ext. 17638, fax: +34 985 10 63 20.

Marta M Pisano-González, Health Service of the Principality of Asturias (SESPA); Oviedo, Spain. Member of the research group 'Atención comunitaria y envejecimiento activo´, Inst Invest Sanitaria Principado Asturias ISPA, Oviedo, Spain.

Verushka Valsecchi, Unité d'Immunologie Clinique et Thérapeutique ostéo-articulaire, Centre Hospitalier Universitaire (CHU) de Montpellier; Montpellier, France.

Siok-Swan Tan, Department of Public Health, Erasmus MC University Medical Center; Rotterdam, the Netherlands.

Yves-Marie Pers, Unité d'Immunologie Clinique et Thérapeutique ostéo-articulaire, Centre Hospitalier Universitaire (CHU) de Montpellier; Montpellier, France.

Raquel Vazquez-Alvarez, Health Service of the Principality of Asturias (SESPA); Oviedo, Spain.

Delia Peñacoba-Maestre, Health Service of the Principality of Asturias (SESPA); Oviedo, Spain.

Graham Baker, Quality Institute for Self Management Education & Training (QISMET); Portsmouth, United Kingdom.

Alberto Pilotto, Department of Geriatric Care, E.O. Galliera Hospital; Genova, Italy.

Sabrina Zora, Department of Geriatric Care, E.O. Galliera Hospital; Genova, Italy.

Hein Raat, Department of Public Health, Erasmus MC University Medical Center; Rotterdam, the Netherlands.

Jose Ramón Hevia-Fernandez, Public Health General Directorate, Ministry of Health of the Principality of Asturias (CSPA); Oviedo, Spain.

On behalf of the EFFICHRONIC Consortium

For peer review only

Word count: 3927

Keywords: Chronic Disease, Social determinants of Health, Caregivers, Patient participation, Self-Management

For peer review only

Abstract

INTRODUCTION

More than 70% of world mortality is due to chronic conditions. Furthermore, it has been proven that social determinants have an enormous impact on both health-related behaviour and on the received attention from healthcare services. These determinants cause health inequalities. The objective of this study is to reduce the burden of chronic diseases in five European regions, hereby focusing on vulnerable populations, and to increase the sustainability of health systems by implementing a chronic disease self-management programme (CDSMP).

METHODS AND ANALYSIS

2,000 people with chronic conditions or informal caregivers belonging to vulnerable populations, will be enrolled in the CDSMP in Spain, Italy, the United Kingdom, France and the Netherlands. Inclusion of patients will be based on geographical, socio-economic and clinical stratification processes. The programme will be evaluated in terms of self-efficacy, quality of life and cost-effectiveness using a combination of validated questionnaires at baseline and 6 months from baseline.

ETHICS AND DISSEMINATION

This study will follow the directives of the Helsinki Declaration and will adhere to the European Union (EU) General Data Protection Regulation (GDPR). The project's activities, progress and outcomes will be disseminated via promotional materials, the use of mass media, online activities, presentations at events and scientific publications.

REGISTRATION DETAILS

The trial was registered at ISRCTN on 20/06/2018 with registry number 70517103.

For peer review only

Article Summary

Strengths and limitations of this study

- Comparable data on the CDSMP implementation in five European countries with different backgrounds will be collected.
- The outcome of the used recruitment strategies, specifically designed for the vulnerable study population, will lead to innovative conclusions.
- The study is not randomized controlled to allow all possible beneficiaries to participate.
- One of the limitations of the study is the fact that vulnerable people, who do not speak or understand the local language, cannot be included.
- Ideally, a longer-term trial should be conducted to measure the outcomes at one-year post-intervention.

For peer review only

Introduction

In the 21st century, chronic conditions are among the most important public health challenges mainly due to their double impact: the human suffering they cause on the one hand and the damage they produce to global socio-economic development on the other. Even though healthcare systems are still primarily focusing on acute diseases[1], they are slowly transforming because of demographic changes, the increasing prevalence of chronic diseases, and the budgetary crisis of recent years. This shift requires new healthcare delivery models to ensure sustainability.

Chronic conditions and also vulnerable settings[2 3] are part of the ten threats to global health the World Health Organization (WHO) has warned of recently. More than 70% of mortality worldwide (41 million people each year), is due to chronic conditions, such as cancer, diabetes, respiratory disorders and heart disease. All of them are related to five main risk factors: tobacco consumption, physical inactivity, alcohol abuse, unhealthy diet and air pollution. Furthermore, 22% of the global population lives in vulnerable settings which exist in almost all regions of the world. As a result, the 13th WHO General Program of Work (2019 – 2023), expresses this concern with the slogan: "Promote health, keep the world safe, serve the vulnerable", urging particularly to address health promotion in populations suffering vulnerability.[3]

The large impact of Non-Communicable Diseases (NCD) on national income is primarily due to loss of productivity caused by absenteeism and inability to work which threaten the sustainability of recent macroeconomic achievements. Additionally, there has been a steady global increase in healthcare expenditure on NCDs over the years. According to a systematic review on healthcare spending and national income, cardiovascular diseases account for 12% of healthcare expenditure in the European Union and diabetes-related costs were estimated at 7%.[4] Besides, other conditions and risks factors such as depression or obesity are becoming more prevalent in higher-income EU countries and are greatly determined by poverty and low income. [5 6]

Also, recent scientific evidence has shown the enormous impact of social determinants (i.e. income, unemployment, housing quality, educational level or gender) on health, which manifest in diverse short- and long-term health inequalities.[7] These differences are not only related to deficient self-care behaviour[1] and poor health, but also to the insufficient attention from healthcare services. Vulnerable people are generally less likely to receive preventive care, timely diagnosis, or follow-up care for their chronic conditions. These observations agree with the 'inverse care law' theory described in 1971[8] by JT Hart, referring to the fact that often, the availability of good medical or social care varies inversely with the need of the population served. This way, chronic conditions and deprivation create a vicious circle.

Fortunately, there is evidence that chronic conditions can be controlled by reducing their risk factors[9], which are mostly linked to unhealthy lifestyle. There is also proof that health literacy programmes that focus on self-efficacy and empowerment can successfully modify many harmful habits. [10 11]

For peer review only - http://bmjopen.bmj.com/site/about/guidelines.xhtml

The basis of the EFFICHRONIC project is the internationally acknowledged Chronic Care Model (CCM)[12] which aims to promote patients' empowerment and autonomy. The specific programme used in the project is the Chronic Disease Self-Management Programme (CDSMP).[13] It concerns a process-centred intervention constructed on the Social Learning Theory[14] and it is appropriate for adults living with one or more chronic conditions. Since its beginning in 1990[15], the effectiveness of the CDSMP has been demonstrated through positive health outcomes and more efficient use of health resources.[16] The programme is community-focused and disseminated through an international infrastructure of training, certification and licensing.

Study Rationale

Up to date, prevention research still concentrates mainly on disease-specific interventions and few studies on particularly vulnerable populations are performed. Moreover, data on the efficiency and cost-benefit of prevention programmes are scarce, which reduces policy engagement and likewise, it prevents health systems from being more sustainable and efficient.[17 18]

EFFICHRONIC aims to empower individuals from (socially) deprived communities to manage their chronic conditions, in an integrated and non-disease specific way. The overall objective of the project is to reduce the burden of chronic conditions and increase the sustainability of health systems by implementing a chronic disease self-management programme in socio-economically vulnerable populations in five European regions.

Five specific objectives have been defined: 1) to identify vulnerable people by means of a multidimensional analysis in the involved regions; 2) to design specific recruitment strategies to reach the target population; 3) to implement the programme in the five regions with at least 400 individuals per region; 4) to generate a comprehensive impact assessment framework, including cost-efficiency and health-economic assessment; and 5) to define guidelines and policy recommendations to allow to the scaling up of the EFFICHRONIC methodology to other regions in Europe.

For peer review only

Methods and analysis

Methodology

The methodology used for the community-based intervention of EFFICHRONIC is the "Chronic Disease Self-Management Programme" developed at Stanford University. The CDSMP is a public health intervention provided by trained monitors. The programme is strongly evidence-based and consists of the following conceptual elements: self-efficacy, empowerment, salutogenesis, peer-to-peer education and vicarious training. It includes action planning and feedback about problem-solving skills and other competencies such as the reinterpretation of symptoms and training in disease management.

A cascade training model is used in the CDSMP method. On top are Self-Management Resource Centre (SMRC) licensed T-trainers possessing the maximum teaching expertise. They are in charge of training Master Trainers, who in their turn train monitors (or leaders) who will conduct the chronic disease self-management workshops. The monitors receive practical training on the philosophical and methodological basis of the CDSMP: empowerment, self-efficacy, group management, management of painful emotions, positive health, and efficient communication among other skills. Once trained, the monitors facilitate the workshops in pairs. In each country, minimum 32 monitors will be trained to instruct the end-users: people with chronic conditions or caregivers. The CDSMP workshops last for 6 weeks with sessions of 2,5 hours once a week in groups of 12 to 20 participants. The programme fidelity and quality plan includes the supervision of workshops in the community by the Master Trainers through periodic protocolled monitor audits. Workshop assistants receive a handout with the workshop topics and homework assignments, a consultation book and a certificate of attendance if they were present at minimum 4 of the 6 sessions.

Study design

EFFICHRONIC is a multicentre non-controlled prospective study with a duration of three years in five European settings: the Principality of Asturias in Spain, the area of Genoa in Italy, several regions in the United Kingdom, the Occitanic region in France and the area of Rotterdam in the Netherlands. The participating countries represent a wide range of social, economic and healthcare-related realities. The EFFICHRONIC intervention consists of the following phases: identification, recruitment and stratification of participants from vulnerable populations, implementation of the intervention and an economic and efficiency evaluation with a pre-post design. Additionally, guidelines and policy recommendations will be developed based on the project conclusions, this to allow the scaling-up of the intervention to other contexts.

Study population

The final study population will consist of 2,000 vulnerable participants distributed over the five implicated areas, who finish the CDSMP (4 out of 6 sessions) and who are evaluated 6 months

For peer review only

after the intervention. Taking into account a drop-out rate of minimum 20%, at least 2,500 participants will be recruited initially.

The vulnerability concept of EFFICHRONIC is based on a definition from the Spanish Red Cross which describes it as “a situation between complete social inclusion and total exclusion”.[19] Keeping in mind this definition and adapting it to some specific requirements of the CDSMP (e.g. being able to understand the local language) and of EFFICHRONIC (being traceable for follow-up at six months), the inclusion criteria were defined (Figure 1).

The target population involves two main groups: vulnerable people with at least one chronic condition, and informal caregivers who are socially isolated. In general, participants must be minimum 18 years old, reside in the selected geographical areas, have their basic housing needs met, and possess adequate knowledge of the local language.

Participants with chronic conditions are either people older than 65 living alone or in a nursing home; people having difficulties to make ends meet (especially if belonging to an ethnic minority or being a legal immigrant, asylum seeker or refugee); or people who are imprisoned. A chronic disease, self-reported or clinically evaluated, is outlined as a pathology listed in the International Classification of Primary Care (ICPC-2) with a code between 70 and 99 in one of the 17 chapters and should have more than six months of evolution.

Informal caregivers are defined as people looking after a sick person, usually a relative, without receiving payment. Additionally, to be included in the study, it is required that they are socially isolated, as is the case of carers living in remote areas without transportation or with limited access to it (without a private car and public transport at more than one kilometre from the house), without internet access or little social support.

General exclusion criteria are: going through a period of crisis (e. g. being evicted), having severe mental health problems that cause a distorted perception of reality and/or inability to participate in group dynamics, having cognitive decline (e.g. Alzheimer´s) or having active addictive disorders. Although the excluded groups are among the most vulnerable of society, they need other and more specific interventions before being eligible for a self-management programme.

Sample size

The study primarily wishes to demonstrate a statistically significant difference in the two subscales of the 12 items Short Form (SF-12)[20] measuring Health-Related Quality of Life, and in the five participating countries. Based on a study on the effect of the CDSMP in lumbar back pain, the sub scores “well-being” and “energy” of the SF-36 improved by 6.0 +/- 19.1 and 4.3 +/- 23.2, respectively after six months.[21] The lowest Cohen’s d is therefore of 0.185 in quality of life domains. Thus, the principal conclusion of the study will rely on 10 tests. Applying the Bonferroni’s correction on the significance threshold, we will consider an alpha risk of 0.005 for the tests measuring the quality of life (self-rated with SF-12) in vulnerable patients with chronic diseases

For peer review only - http://bmjopen.bmj.com/site/about/guidelines.xhtml

and caregivers. To show a statistically significant effect size of 0.185, by a paired Student's T-test, with a bilateral alpha risk of 0.005, a power of 0.9, and a correlation between baseline and 6 months of 0, we need to analyze 978 subjects. Assuming a drop-out rate of 20%, we need to include at least 1223 subjects. The multicentre study will include 2,000 subjects (400 per country), which will bring about sufficient power to analyze the objectives.

Sample selection and recruitment

The potential participants are being identified by means of geographical, socio-economic and clinical stratification processes. A distinction is made between the individual and social dimension of vulnerability, leading to different recruitment strategies that will be combined to be more efficient. First, geographical mapping of areas with a higher percentage of vulnerable population was done based on existing national and local indices in all participating countries. Consecutively, individualized recruitment will take place in these areas employing specifically designed actions. Multiple recruitment channels are used in coordination with local agents from the healthcare system, social services and community-based structures. More specifically, the following profiles and organizations are involved in recruitment: health professionals, pharmacists, social workers, Non-Governmental Organisations (NGOs), city councils and associations. The recruitment strategy at each study site is adapted to the specific local circumstances and collaborating organizations.

Stratification

To identify the people who will benefit most from the programme, individual socio-economic and clinical parameters will be measured by a multidimensional stratification analysis. The tool used for this purpose, the "SELFY-MPI", has been developed based on the validated Multidimensional Prognostic Index (MPI).[22] The new tool is self-administrable and was specifically designed for EFFICHRONIC,[23] adapting it to the broad adult population (not limited to the elderly), and incorporating the socio-economic parameters from the Spanish Gijón scale: education, income, co-habitation, age, and social support. [24] Income categories are country-specific and built on the corresponding minimum wages.

Implementation

Once the target population is identified and recruitment is ongoing, the programme can be implemented. To reach the target sample size of 2,000 people, between 100 and 140 workshops (20 to 28 per country) will have to be conducted. In practice, for about a year and a half, one workshop will start every month at each study site.

Evaluation

The last phase of the project consists of evaluating the effectiveness of the programme when it comes to changing health-related behaviour, self-efficacy and quality of life. Furthermore, the economic and efficiency impact will be analyzed through cost-benefit analyses. An auto-administrable impact questionnaire, containing validated scales and measuring tools, has been

For peer review only

designed to assess these different outcome measures at baseline (T0, before the CDSMP) and 6 months (T1).[25]

Statistical analysis plan

Differences between baseline and follow-up measurements will be assessed employing the paired T-test (for normally distributed variables), the Mann Whitney U test (for variables not normally distributed) and the Pearson Chi-square test (for variable fractions). To adjust for multiple testing, one-way analyses of variance with post hoc testing (type Bonferroni) will be performed. The relationship of an outcome measure (T1) with explanatory variables will be determined using ordinary least squares (OLS) regression analysis. The outcome measure (T0), (change in) other outcome measures, risk factors and social determinants may serve as explanatory variables. Additionally, the above-mentioned analyses will be repeated for each country separately and possibly for other subgroups (for variables which are likely to influence the effect of the intervention itself, e.g. age and gender). All data from each of the five pilot sites will be anonymized and centralized at one study site for statistical analysis. Analyses will be performed with SPSS version 25.0 (IBM SPSS Statistics for Windows, Armonk, NY: IBM Corp).

Guidelines and Policy recommendations

By the end of the project, guidelines and policy recommendations will be formulated based on the specific results of the participating countries. These recommendations will take into account acknowledged Best Practices at European level, to allow the scaling up of community-based interventions in vulnerable populations.

Patient and public involvement

Patients were not involved in the design of the study, choice of outcome measures or definition of the recruitment strategies. However, some patients and caregivers take an active part in the conduct of the study as trained monitors, facilitating workshop sessions in the community. Study participants will be able to access the study results on request as stated in the participant information letter. News on the study progress is being published via various general and specialized media channels throughout the running of the study.

For peer review only

Ethics and dissemination

It is not considered ethical to conduct descriptive research on vulnerable subjects without running specific interventions afterwards. For this reason, the study is not randomised-controlled, being consistent with the principle of social justice and the ethics of working with marginalized groups. [26]

The study follows the directives of the Helsinki declaration and the corresponding ethical regulations are being respected at each study site. In Occitanie, France, the Ethics Committee of the South-west and Oversees I in Toulouse (Comité de Protection des Personnes Sud-Ouest et Outre-Mer I) approved the study on 05/11/2018; study number 9788. In Genoa, Italy, the Regional Ethics Committee of Liguria (Il Comitato Etico della Regione Liguria) approved the study on 27/03/2018; study number 152-2018. In Rotterdam, the Netherlands, the Medical Ethics Review Committee (Medische Ethische Toetsings Commissie; METC) of the Erasmus MC University Medical Center - Rotterdam approved the study on 23/11/2017; study number MEC-2017-1116.
In Asturias, Spain, the Research Ethics Committee of the Principality of Asturias (Comité de Ética de la Investigación del Principado de Asturias) approved the study on 31/01/2017; study number 20/17. In the United Kingdom, the online decision tool and query line of the Health Research Authority were consulted and it was concluded that approval from the NHS Research Ethics Committee was not necessary.

Eligible participants will be informed of the objectives and the procedures of the CDSMP in person by the investigator and in writing by a participant information letter before enrolment. Only after a sufficient period of reflection and after clarification of all questions, the participant will be asked to sign two copies of the informed consent form. Participation in the study is completely voluntary and participants have the right to withdraw their consent from the study at any time for any reason.

Data processing, communication and transfer are done following the EU Regulation 2016/679 of the European Parliament and of the Council of 27 April 2016, also known as the GDPR. [27] Only the research team that has to maintain confidentiality has access to all collected study data. Only impersonalised information may be transmitted to third parties. Information transmission to other countries will be carried out anonymously with a data protection level according to the mentioned regulation. The data will be collected and preserved in a codified way until the study is finished. The person responsible for data custody is responsible for the data file and treatment. At the end of the study, the data will be anonymized.

Via promotional materials, use of mass media, online activities, presentations at events and scientific publications, the project's activities, progress and outcomes will be disseminated as described in a specific communication and dissemination plan. The strategy aims to enhance the engagement of healthcare and social care providers, civil society and public administration officers, all of which multipliers on account of their broad network and potential influence on the target population. Nevertheless, the main intended beneficiaries of the strategy are vulnerable

For peer review only - http://bmjopen.bmj.com/site/about/guidelines.xhtml

people with long-term conditions and the health services serving these people. Accessing the hard-to-reach is one of the major challenges in EFFICHRONIC for which a solid strategy will be used.

For peer review only

Author contributions

MMPG designed the initial study protocol. ALDB, VV, SST, YMP, RVA, DPM, GB, AP, SZ, HR and JRHF advised on the study protocol. ALDB and MMPG drafted the manuscript. All authors critically revised the manuscript on important content and approved the final manuscript.

Funding statement

This work was supported by the European Union's Health Programme (2014-2020) grant number 738127.

Competing interests statement

The authors have no competing interests to declare.

Acknowledgements

The members of the EFFICHRONIC Consortium are: Marta M Pisano Gonzalez, Raquel Vazquez Alvarez, Delia Peñacoba Maestre (Health Service of the Principality of Asturias – SESPA, Spain); An L. D. Boone, José Ramón Hevia Fernandez and Sergio Valles García (Ministry of Health of the Principality of Asturias – CSPA, Spain); Sonia López-Villar, Inés Rey Hidalgo and Raquel Ochoa Gonzalez (the Foundation for the Promotion in Asturias of applied Scientific Research and Technology – FICYT, Spain); Yves-Marie Pers, Christian Jorgensen, Verushka Valsecchi, Rosanna Ferreira, Adrien Durand, Cristina Balaguer Fernandez, Dallal Fracso (The University Hospital Center of Montpellier – CHUM, France); Graham Baker, Danni Brown and Suzanne Lucas (Qismet, Portsmouth, UK); Siok-Swan Tan, Irene Fierloos, Xuxi Zhang, Petra de Vries, Hein Raat (Erasmus MC University Medical Center, Rotterdam, The Netherlands); Alberto Pilotto, Sabrina Zora, Alberto Ferri, Alberto Cella and Alessandra Argusti (EO Galliera Hospital, Genua, Italy); Ascensión Doñate Martínez, Laura Llop Medina and Jorge Garcés (University of Valencia, Polibienestar Research Institute – UVEG, Valencia, Spain).

The authors would like to thank reviewer Mr Cesar Fernandez Lazaro for his critical reading and valuable suggestions for improvement, Sabine Talloen for her thorough revision and language editing, and Rosa Carretero de Lama for her administrative support.

Figure legend

Figure 1: EFFICHRONIC: inclusion criteria flow chart

For peer review only

REFERENCES

[1] Barnett K, Mercer S, Norbury M, *et al*. Epidemiology of multimorbidity and implications for health care, research, and medical education: a cross-sectional study. *The Lancet* 2012; 380(9836): 37-43.
[2] World Health Organization [Internet]. Geneva; c2019 [cited 2019 Feb 13] [1 screen]. Available from: https://www.who.int/emergencies/ten-threats-to-global-health-in-2019.
[3] World Health Organization [Internet]. Geneva; c2018 [cited 2019 Feb 13] [pdf file]. Available from: http://apps.who.int/gb/ebwha/pdf_files/WHA71/A71_4-en.pdf.
[4] Muka T, Imo D, Jaspers L, *et al*. The global impact of non-communicable diseases on healthcare spending and national income: a systematic review. *Eur J Epidemiol* 2015; 30:251–277.
[5] Dalstra JA, Kunst AE, Borrell C, *et al*. Socioeconomic differences in the prevalence of common chronic diseases: an overview of eight European countries. *International journal of epidemiology 2005; 34*(2): 316-326.
[6] Kontis V, Mathers CD; Rehm J, *et al*. Contribution of six risk factors to achieving the 25× 25 non-communicable disease mortality reduction target: a modelling study. *The Lancet* 2014; *384*(9941): 427-437.
[7] Marmot M, Wilkinson R. Social determinants of health. Oxford: *Oxford University Press*, 2006.
[8] Hart J. The inverse care law. *The Lancet* 1971*; 297*(7696): 405-412.
[9] Bauer UE, Briss PA, Goodman RA, *et al*. Prevention of chronic disease in the 21st century: elimination of the leading preventable causes of premature death and disability in the USA. *The Lancet* 2014*;* 384(9937): 45-52.
[10] Bodenheimer T, Lorig K, Holman H, *et al*. Patient self-management of chronic disease in primary care. *Jama* 2002*; 288*(19): 2469-2475.
[11] Smith ML, Wilson MG, Robertson MM, *et al*. Impact of a Translated Disease Self-Management Program on Employee Health and Productivity: Six-Month Findings from a Randomized Controlled Trial**.** *Int J Environ Res Public Health* 2018; 15(5): 851.
[12] Barr VJ, Robinson S, Marin-Link B *et al*. The expanded Chronic Care Model: an integration of concepts and strategies from population health promotion and the Chronic Care Model. *Healthcare Quarterly* 2003; *7*(1).
[13] Lorig K, Sobel DS, Ritter PL, *et al*. Effect of a self-management program on patients with chronic disease. *Eff. Clin. Pract*. 2001;4:256–262.
[14] Bandura A. Social cognitive theory of self-regulation. *Organ. Behav. Hum. Decis. Process.* 1991; 50: 248–287.
[15] Lorig K, Sobel DS, Stewart AA, *et al*. Evidence suggesting that a chronic disease self-management program can improve health status while reducing hospitalization: A randomized trial. *Med. Care.* 1999;37:5–14.
[16] Ory MG, Smith ML, Kulinski KP, *et al*. Self-management at the tipping point: Reaching 100,000 Americans with evidence-based programs. *J. Am. Geriatr. Soc*. 2013;61:821–823.
[17] Harrell JS, Bangdiwala SL, Deng S. Smoking initiation in youth: the roles of gender, race, socioeconomics, and developmental status. *Journal of Adolescent Health* 1998; *23*(5): 271-279.
[18] Porter SN. Poverty, Discrimination, and Health. *Social Pathways to Health Vulnerability* 2019; 23-53.
[19] Malgesini RG, Rua VA, Martinez IZ. Informe sobre la Vulnerabilidad Social. *Cruz Roja Española* 2015; 1-120.
[20] Ware J, Kosinski M, Keller SD. A 12-Item Short-Form Health Survey: construction of scales and preliminary tests of reliability and validity. *Med Care* 1996; 34(3):220-233.

[21] Haas M, Groupp E, Muench J, *et al*. Chronic Disease Self-Management Program for Low Back Pain in the Elderly. *Journal of Manipulative and Physiological Therapeutics* 2005; 28 (4): 228-237.

[22] Pilotto A, Ferrucci L, Franceschi M, *et al*. Development and validation of a multidimensional prognostic index for one-year mortality from comprehensive geriatric assessment in hospitalized older patients. *Rejuvenation research* 2018; *11*(1): 151-161.

[23] Pilotto A, Veronese N, Quispe K, *et al*. Development and validation of a self-administered Multidimensional Prognostic Index (SELFY-MPI) to predict negative health outcomes in community-dwelling persons. *Rejuvenation research* 2019; 22(4): 299-305.

[24] Garcia-Caselles P, Miralles R, Arellano M ,*et al.* Validation of a modified version of the Gijon's social-familial evaluation scale (SFES): The "Barcelona SFES Version", for patients with cognitive impairment. *Arch. Gerontol. Geriatr. Suppl.* 2004; 9: 201–206.

[25] Tan SS, Pisano MM; Boone AL, et al. Evaluation Design of EFFICHRONIC: The Chronic Disease Self-Management Programme (CDSMP) Intervention for Citizens with a Low Socioeconomic Position. Int. J. Environ. Res. Public Health 2019; 16 (11): 1883.

[26] Mosavel M, Simon C. Exploratory Health Disparities Research: The Need to Provide a Tangible Benefit to Vulnerable Respondents. *Ethics & Behavior* 2010; 20(1), 1–9.

[27] European Union Law [internet] [cited 2019 Feb 13] [1 screen]. Available from https://eur-lex.europa.eu/eli/reg/2016/679/oj.

For peer review only

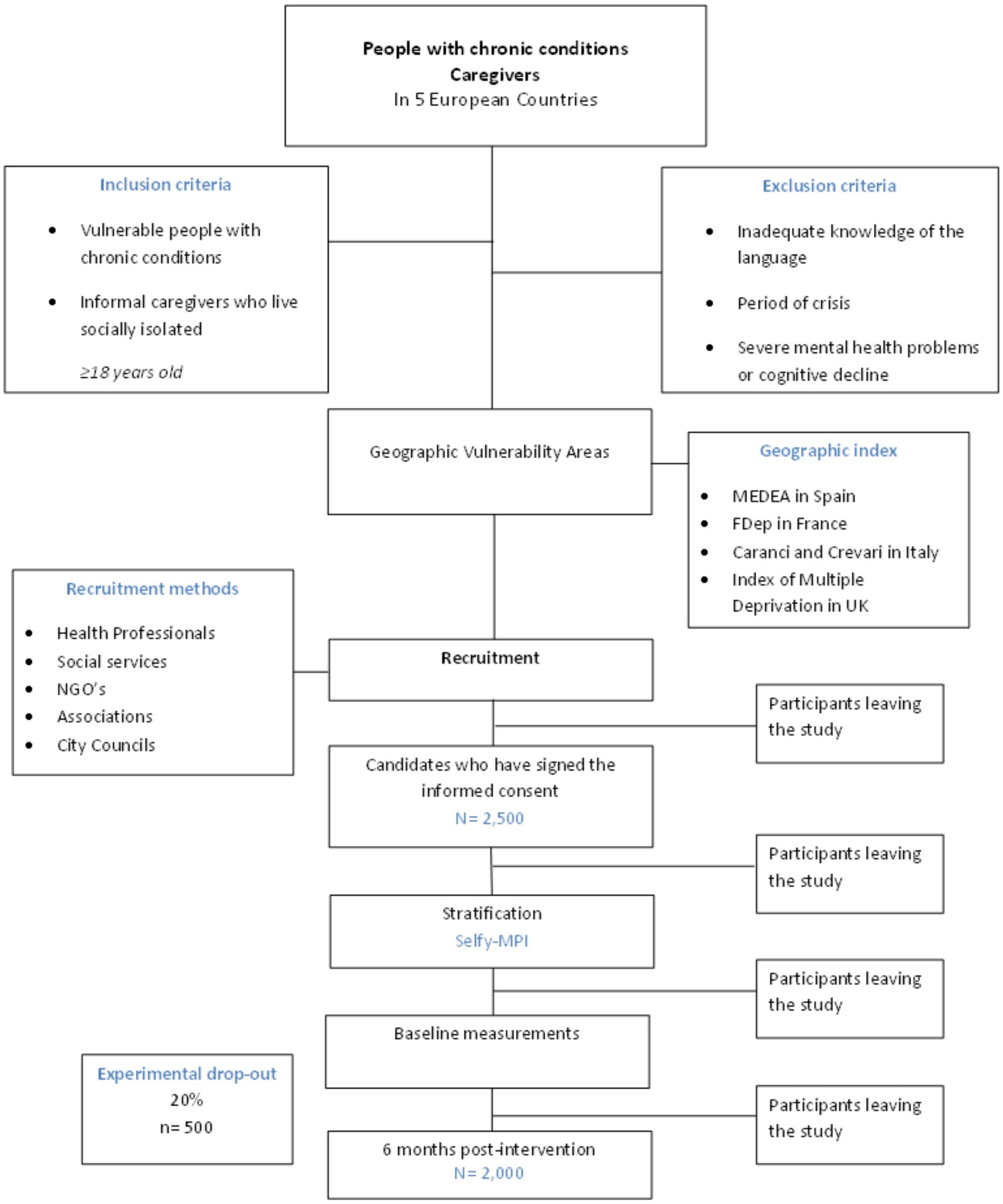

EFFICHRONIC: inclusion criteria flow chart

64x77mm (300 x 300 DPI)
